# Dasabuvir Inhibits Human Norovirus Infection in Human Intestinal Enteroids

**DOI:** 10.1128/mSphere.00623-21

**Published:** 2021-11-03

**Authors:** Tsuyoshi Hayashi, Kosuke Murakami, Junki Hirano, Yoshiki Fujii, Yoko Yamaoka, Hirofumi Ohashi, Koichi Watashi, Mary K. Estes, Masamichi Muramatsu

**Affiliations:** a Department of Virology II, National Institute of Infectious Diseases, Tokyo, Japan; b Department of Applied Biological Science, Tokyo University of Science, Chiba, Japan; c Research Center for Drug and Vaccine Development, National Institute of Infectious Diseases, Tokyo, Japan; d Departments of Molecular Virology and Microbiology and of Medicine, Baylor College of Medicine, Houston, Texas, USA; University of Maryland School of Medicine

**Keywords:** acute gastroenteritis, antiviral drug, compound screen, dasabuvir, intestinal enteroids, norovirus

## Abstract

Human noroviruses (HuNoVs) are acute viral gastroenteritis pathogens that affect all age groups, yet no approved vaccines and drugs to treat HuNoV infection are available. In this study, we screened an antiviral compound library to identify compound(s) showing anti-HuNoV activity using a human intestinal enteroid (HIE) culture system in which HuNoVs are able to replicate reproducibly. Dasabuvir (DSB), which has been developed as an anti-hepatitis C virus agent, was found to inhibit HuNoV infection in HIEs at micromolar concentrations. Dasabuvir also inhibited severe acute respiratory syndrome coronavirus 2 (SARS-CoV-2) and human rotavirus A (RVA) infection in HIEs. To our knowledge, this is the first study to screen an antiviral compound library for HuNoV using HIEs, and we successfully identified dasabuvir as a novel anti-HuNoV inhibitor that warrants further investigation.

**IMPORTANCE** Although there is an urgent need to develop effective antiviral therapy directed against HuNoV infection, compound screening to identify anti-HuNoV drug candidates has not been reported so far. Using a human HIE culture system, our compound screening successfully identified dasabuvir as a novel anti-HuNoV inhibitor. Dasabuvir’s inhibitory effect was also demonstrated in the cases of SARS-CoV-2 and RVA infection, highlighting the usefulness of the HIE platform for screening antiviral agents against various viruses that target the intestines.

## INTRODUCTION

Human noroviruses (HuNoVs) cause acute gastroenteritis and foodborne diseases among all age groups worldwide. HuNoVs often cause an economic burden to societies due to health care costs and loss of productivity and therefore pose a public health concern. Noroviruses are nonenveloped viruses possessing a positive-sense, single-stranded RNA genome whose length is approximately 7.5 kb. They are genetically classified in 10 genogroups (GI to GX) and further divided into 48 genotypes based on their capsid and polymerase gene sequences (1). Among those, the GII.4 genotype is the most frequently distributed and causes outbreaks in humans worldwide (2, 3).

Since a robust culture system to allow HuNoV replication was not established for almost 50 years, there are no established antiviral regimens or vaccines available. Recently, several HuNoV successive cultivation models employing a human B-cell line (4), tissue stem cell-derived human intestinal enteroids (HIEs) (5), human induced pluripotent stem cell-derived intestinal organoids (6), and zebrafish larvae (7) have been developed. The stem cell-derived HIE system is currently used by researchers worldwide to study HuNoV biology and viral inactivation strategies (8–14), although, to our knowledge, compound screens for identifying HuNoV antiviral agents have not been reported previously.

Drug repurposing is a time-saving, affordable strategy to discover new therapeutic uses for approved or developing drugs to treat other disease(s) apart from their original use(s) (15). This strategy is being widely utilized to establish effective therapeutics for the treatment of coronavirus disease 2019 (COVID-19). Indeed, numerous antiviral drugs, including remdesivir, ivermectin, or nelfinavir have been identified as promising candidates against severe acute respiratory syndrome coronavirus 2 (SARS-CoV-2) (15, 16). Here, with the HIE culture system, we screened an antiviral compound library composing 326 bioactive substances, including those targeting influenza virus, human immunodeficiency virus (HIV), or hepatitis C virus (HCV) to reassess their effect on HuNoV infection.

## RESULTS AND DISCUSSION

**FIG 1:**
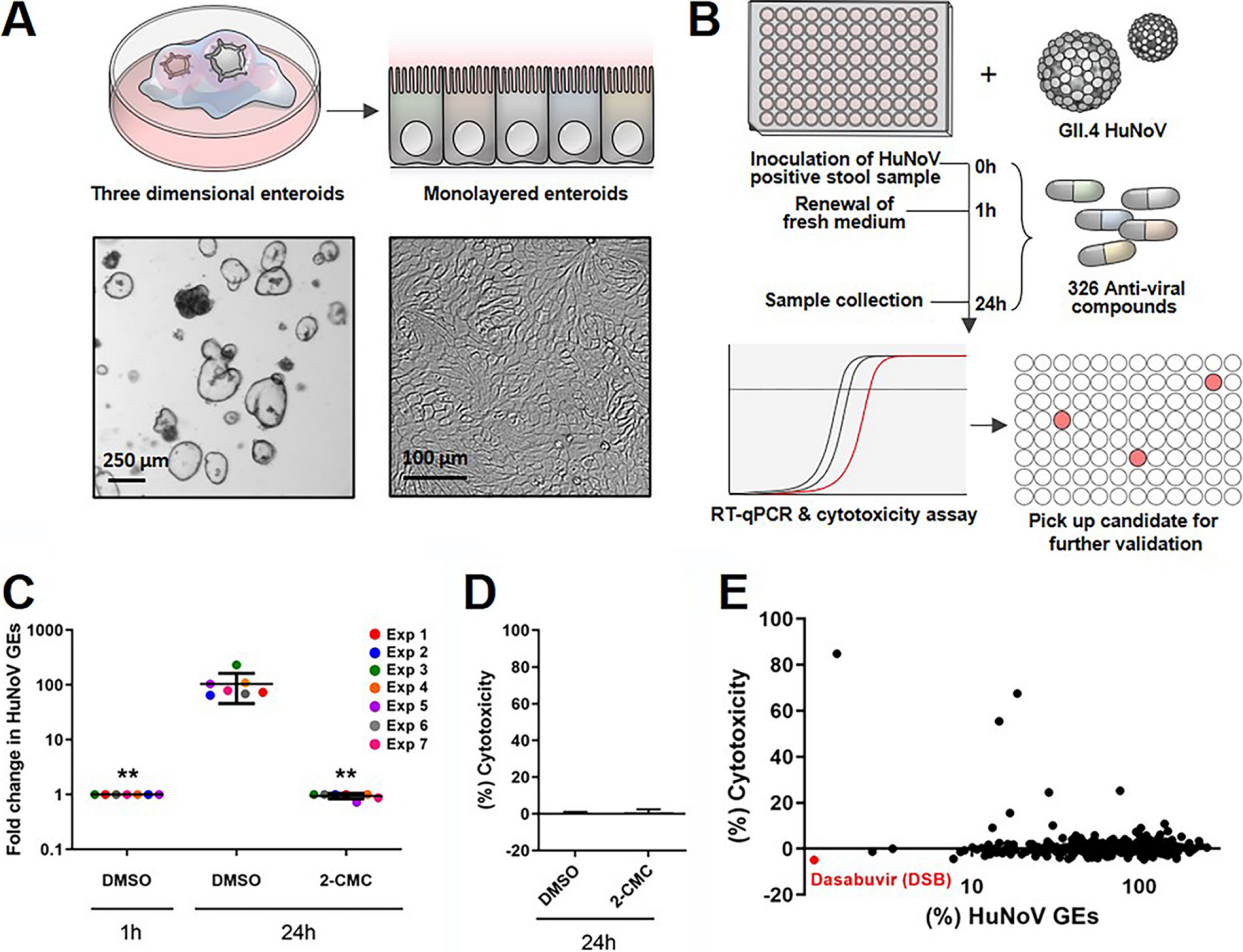
Screening for antiviral compounds that inhibit human norovirus (HuNoV) infection in human intestinal enteroids (HIEs). (A and B) Schematic illustrations of compound screening. Three-dimensional HIEs (J2) were dissociated into single cells using TrypLE enzyme and plated in a 96-well plate to culture them as 2D monolayers. Differentiated HIE monolayers were then inoculated with GII.4 HuNoV-containing stool filtrates in the presence of the compounds (10 μM, *n* = 1). After 1 h incubation at 37°C, the cells were washed and then cultured in differentiation medium containing the compounds (10 μM) in a 100-μl volume until 24 h postinfection (hpi). Viral RNA extracted from the cells and 75 μl of supernatant at 1 or 24 hpi were subjected to reverse transcription-quantitative PCR (RT-qPCR) to measure viral genome equivalents (GEs). The rest of the supernatant was subjected to lactate dehydrogenase (LDH) assay to measure cytotoxicity. (C) HuNoV replication in J2 monolayers throughout the screening. We performed 7 experiments to screen all 326 compounds. Dimethyl sulfoxide (DMSO) and 2′-C-methylcytidine (2-CMC; 389 μM) were used as the controls in every test. Viral GEs in DMSO and 2-CMC-treated samples at 24 hpi were normalized to the DMSO control at 1 hpi. **, *P < *0.01 versus DMSO control at 24 hpi, one-way analysis of variance (ANOVA) followed by Dunnett's multiple-comparison test. (D) Cytotoxicity of 2-CMC in J2 monolayers at 24 hpi. Results were normalized to the DMSO control. (E) Scatterplot of the % HuNoV GEs versus % cytotoxicity for all tested compounds. Results were normalized to the DMSO control. DSB, dasabuvir.

Three-dimensional (3D) HIEs were dissociated and plated on collagen-coated 96-well plates to prepare two-dimensional (2D) monolayers (Fig. 1A). The cells were then differentiated by culturing them in differentiation medium, which does not include Wnt3A and R-spondin to support the stemness of HIEs. The differentiated HIE monolayers were inoculated with GII.4 HuNoV in the presence of each compound dissolved in dimethyl sulfoxide (DMSO) for 1 h at 37°C. DMSO was added to the wells without compound (DMSO control). For this screening step, one well was used to analyze each compound (*n* = 1). The cells were washed, then cultured in differentiation medium containing the compound for 24 h. The infected cells and supernatant were then harvested, and viral replication was evaluated by reverse transcription-quantitative PCR (RT-qPCR) analysis to determine the HuNoV RNA genome equivalents (GEs) (Fig. 1B). Cytotoxicity for each compound was also determined by lactate dehydrogenase (LDH) assay. First, to evaluate the reproducibility of our HIE system with respect to HuNoV growth throughout the screening, we plotted the level of viral GEs at 1 or 24 h postinfection (hpi) from 7 independent experiments that were used for the compound screen. The fold changes of viral GEs between 1 and 24 hpi in each experiment ranged from 65 to 230 (mean ± standard deviation [SD], 104.3 ± 58.3). A positive control, 2′-C-methylcytidine (2-CMC), completely blocked viral infection without any cytotoxicity in all experiments, consistent with the results of a previous report (Fig. 1C and D) (11). These results demonstrate that our HIE cultivation system reproducibly supports HuNoV replication and is suitable for evaluating the effect of the compounds against HuNoV.

We next determined the relative percentages of HuNoV GE and cytotoxicity at 24 hpi by normalizing the data of compound-treated cells to that of DMSO-treated cells. The screening results were plotted as HuNoV GE (%) versus cytotoxicity (%) (Fig. 1E). Among 326 compounds tested, dasabuvir (DSB) showed the strongest inhibitory effect on HuNoV infection without compromising cell viability (Fig. 1E and Table S1 in the supplemental material). We then repeated the experiment with 3 technical replicates and confirmed its inhibitory effect against HuNoV infection (Fig. S1). DSB has been developed as a direct-acting anti-HCV drugs that targets HCV NS5B RNA-dependent RNA polymerase (RdRp) (17). So far, there have been no reports regarding its antiviral effects against HuNoV.

10.1128/mSphere.00623-21.1 FIG S1 Validation of dasabuvir (DSB) in J2 human intestinal enteroid (HIE) monolayers. J2 HIE monolayers were inoculated with GII.4 human norovirus (HuNoV)-containing stool filtrate in the presence of dasabuvir (10 μM) and were cultured until 24 hpi. The percentages of HuNoV genome equivalents (GEs) (A) and cytotoxicity (B) for each compound were determined as in Fig. 1. Values represent the mean ± standard deviation (SD) (dimethyl sulfoxide [DMSO] control at 1 hpi, *n* = 2; DMSO control at 24 hpi, *n* = 4; DSB at 24 hpi, *n* = 3). **, *P < *0.01 versus DMSO control at 24 hpi, two-tailed Student’s *t* test. (C) Chemical structure of dasabuvir. Download FIG S1, TIF file, 1.5 MB. Copyright © 2021 Hayashi et al. 2021 Hayashi et al. https://creativecommons.org/licenses/by/4.0/ This content is distributed under the terms of the Creative Commons Attribution 4.0 International license.

10.1128/mSphere.00623-21.5 TABLE S1 Compound screening results. The percentages of human norovirus (HuNoV) genome equivalents (GEs) and cytotoxicity in the presence of the indicated compound (10 μM, *n* = 1) were determined and normalized to those of dimethyl sulfoxide (DMSO) control. Download Table S1, XLSX file, 0.02 MB. Copyright © 2021 Hayashi et al. 2021 Hayashi et al. https://creativecommons.org/licenses/by/4.0/ This content is distributed under the terms of the Creative Commons Attribution 4.0 International license.

**FIG 2:**
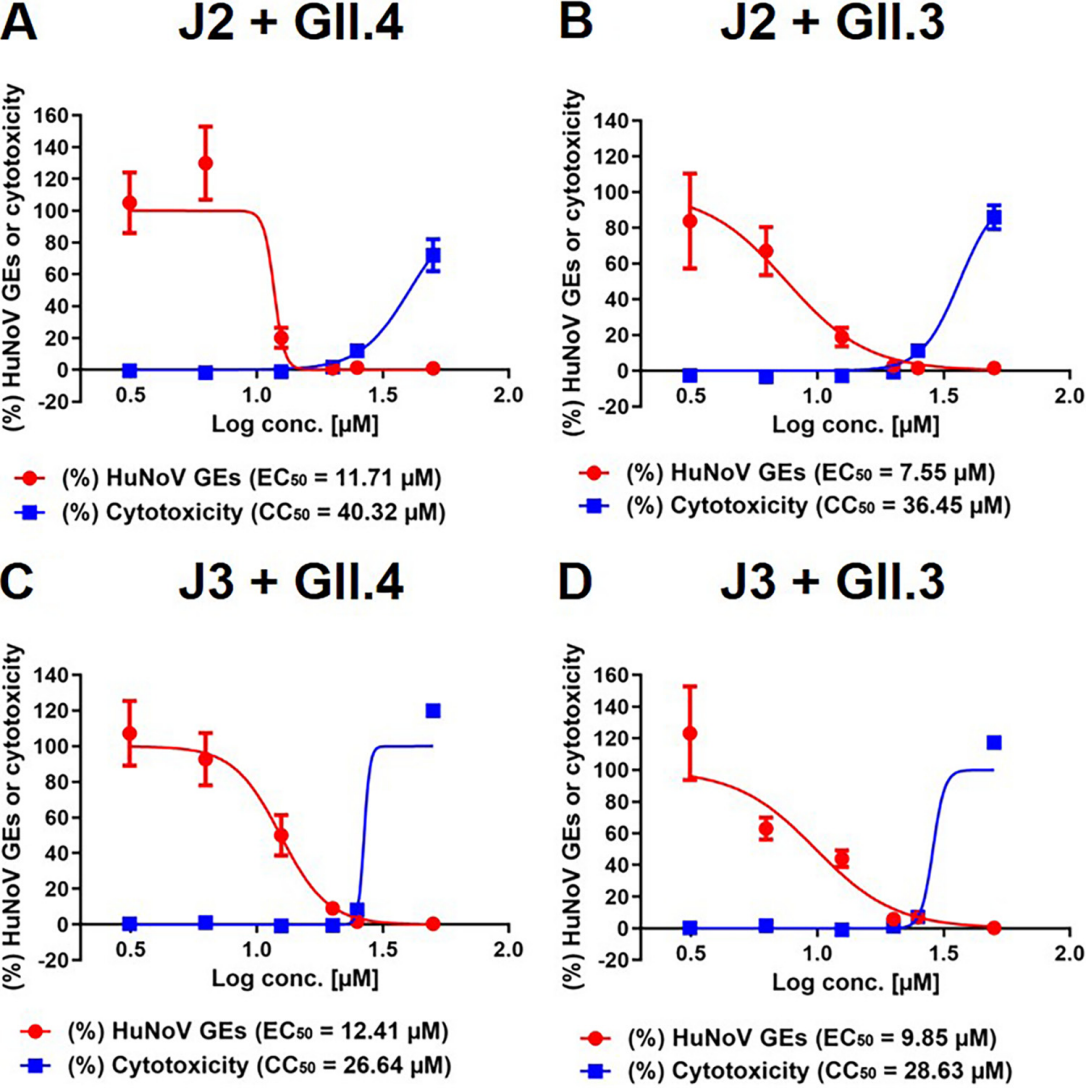
Effect of dasabuvir on HuNoV infection in HIE monolayers. J2 (A and B) or J3 (C and D) HIE monolayers were inoculated with GII.4 (A and C) or GII.3 (B and D) HuNoV-containing stool filtrate in the presence of DSB at the indicated concentrations and were cultured until 20 hpi. The percentages of HuNoV GEs (red lines) and cytotoxicity (blue lines) were determined as in Fig. 1 and were normalized to the DMSO control. Values represent the mean ± standard deviation (SD) (*n* ≥ 6). EC_50_, 50% effective concentration; CC_50_, 50% cytotoxic concentration.

Next, we performed additional experiments with DSB at various concentrations ranging from 3.125 μM to 50 μM to calculate the 50% effective concentration (EC_50_) and 50% cytotoxic concentration (CC_50_) values. DSB treatment alone showed no cytotoxicity, except for the highest concentration (50 μM), which showed a 17% reduction of cellular ATP (cell viability) or a 10% increase of LDH release (cytotoxicity), compared to those of the DMSO control (Fig. S2). Again, DSB did not induce cytotoxicity, except at the highest dose (50 μM), in J2 monolayers infected with GII.4 HuNoV, whereas it showed a dose-dependent inhibition of viral replication with an EC_50_ value of 11.71 μM (Fig. 2A). To ascertain the authenticity of DSB’s inhibitory effect, we repeated the experiment with the identical compound from different sources and found that the results were comparable (Fig. S3). Dose-dependent reduction of viral replication by DSB was also observed in J2 HIE monolayers infected with a different HuNoV strain GII.3 (Fig. 2B). We further assessed DSB’s inhibitory effect using J3 HIEs established from an independent donor following infection with GII.3 or GII.4 HuNoV and observed the same trends (Fig. 2C and D). Taken together, DSB exerted an inhibitory effect on two HuNoV genotypes and HIEs established from distinct individuals, suggesting that this effect is likely to be neither genotype nor HIE (donor) dependent.

10.1128/mSphere.00623-21.2 FIG S2 Cell viability and cytotoxicity of dasabuvir in J2 HIE monolayers. J2 HIE monolayers were treated with DSB at the indicated concentrations for 20 h. Cell viability or cytotoxicity was measured using CellTiter-Glo luminescent cell viability assay or the Cytotoxicity LDH assay kit-WST, respectively. Results were normalized to DMSO control. Values represent the mean ± SD (*n* = 7). Download FIG S2, TIF file, 0.8 MB. Copyright © 2021 Hayashi et al. 2021 Hayashi et al. https://creativecommons.org/licenses/by/4.0/ This content is distributed under the terms of the Creative Commons Attribution 4.0 International license.

10.1128/mSphere.00623-21.3 FIG S3 Inhibitory effect of dasabuvir purchased from different sources on HuNoV infection in J2 HIE monolayers. J2 HIE monolayers were inoculated with GII.4 HuNoV-containing stool filtrate in the presence of dasabuvir (DSB) purchased from different sources at the indicated concentrations and cultured until 20 hpi. The percentages of HuNoV GEs and cytotoxicity were determined and were normalized to the DMSO control. Values represent the mean ± SD (*n* = 4). Download FIG S3, TIF file, 1.3 MB. Copyright © 2021 Hayashi et al. 2021 Hayashi et al. https://creativecommons.org/licenses/by/4.0/ This content is distributed under the terms of the Creative Commons Attribution 4.0 International license.

**FIG 3:**
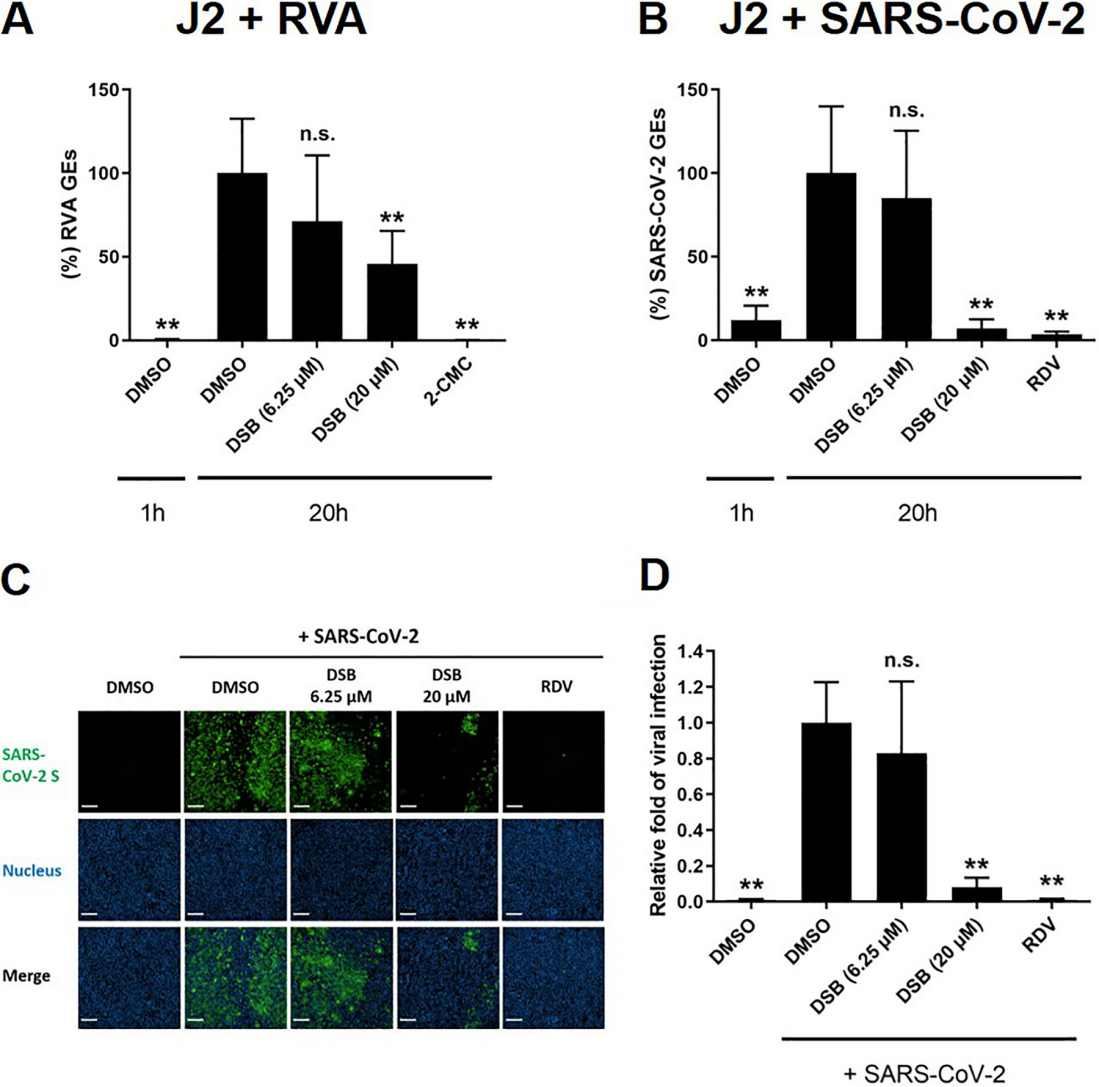
Dasabuvir inhibits SARS-CoV-2 infection in J2 HIE monolayers and VeroE6/TMPRSS2 cells. (A and B) J2 HIE monolayers were inoculated with RVA (A) and SARS-CoV-2 (B) in the presence of the indicated compounds and were cultured until 20 hpi. 2-CMC (389 μM) or remdesivir (RDV; 10 μM) were used as positive controls. The percentages of viral GEs were determined by RT-qPCR, and were normalized to the DMSO control at 20 hpi. Values represent the mean ± SD (*n* ≥ 5). (C and D) VeroE6/TMPRSS2 cells were left uninfected or were infected with SARS-CoV-2 for 20 h in the presence of the indicated compounds. The cells were then stained with anti-SARS-CoV-2 spike (S) RBD monoclonal antibody and 4′,6-diamidino-2-phenylindole (DAPI), followed by imaging analysis. (C) Representative fluorescence images showing SARS-CoV-2 S protein (green) and cell nucleus (blue). Bar, 200 μm. (D) The percentages of infected cells were normalized to those of DMSO-treated cells infected with SARS-CoV-2 at 20 hpi. Values represent the mean ± SD (*n* ≥ 8). **, *P < *0.01 versus DMSO control at 20 hpi, one-way ANOVA followed by Dunnett’s multiple-comparison test. n.s., not significant (*P > *0.05).

Next, we tested the effect of DSB on infection with human rotavirus A (RVA) and SARS-CoV-2, both of which has been previously reported to be able to infect and replicate HIEs (18–20). Two concentrations of DSB were used in these studies, namely, noneffective (6.25 μM) and effective concentration (20 μM) against HuNoV. As shown in Fig. 3A and B, DSB showed a moderate antiviral effect (2.75-fold decrease) on RVA infection at a concentration of 20 μM, while it almost completely inhibited (26.9-fold decrease) SARS-CoV-2 infection in J2 HIE monolayers. We also confirmed DSB’s inhibition with no cytotoxicity using VeroE6/TMPRSS2 cells, which are highly susceptible to SARS-CoV-2 infection (Fig. 3C and D, and Fig. S4) (21).

10.1128/mSphere.00623-21.4 FIG S4 Cell viability of dasabuvir in VeroE6/TMPRSS2 cells. VeroE6/TMPRSS2 cells were left uninfected or were infected with SARS-CoV-2 for 20 h, followed by immunofluorescence analysis, as described in Materials and Methods in the main text. The cell numbers were determined by counting cell nuclei. Results were normalized to DMSO-treated cells infected with SARS-CoV-2. Values represent the mean ± SD (*n* = 8). Download FIG S4, TIF file, 0.9 MB. Copyright © 2021 Hayashi et al. 2021 Hayashi et al. https://creativecommons.org/licenses/by/4.0/ This content is distributed under the terms of the Creative Commons Attribution 4.0 International license.

The mechanism of action for the virus inhibitions by DSB remains to be elucidated. DSB is a nonnucleotide inhibitor of HCV NS5B RdRp that likely binds to the palm domain of NS5B and thereby prevents elongation of the nascent viral genome (22). Therefore, it might also target the RdRp of other viruses, such as HuNoV and SARS-CoV-2, possibly because of the presence of conserved sequences being targeted by DSB. Indeed, there is a report showing that DSB partially inhibits the RdRp activity of Middle East respiratory syndrome coronavirus (MERS-CoV) (23). Targeting of viral protease might be another scenario for the inhibition; a very recent virtual screening study predicted that dasabuvir has the potential to inhibit 3-chymotrypsin-like protease (3CL^PRO^) of SARS-CoV-2 (24).

With an HCV subgenomic replicon system, DSB inhibits HCV of genotype 1 with EC_50_ values of <10 nM (22). In contrast, DSB inhibits HuNoV infection with EC_50_ values ranging between 7.55 and 12.41 μM (Fig. 2), which is comparable to its effectiveness in inhibiting RdRp activity of MERS-CoV-2 (23) or infection by vector-borne flaviviruses (25). This implies that a higher concentration is required to exert an antiviral effect on non-HCV viruses, possibly due to lower binding efficiency of DSB to non-HCV RdRp(s) or to unknown mechanism(s) of its inhibitory action. A pharmacokinetic study of DSB in healthy volunteers demonstrated that the maximum plasma concentration (*C*_max_) is reached at approximately 5.61 μM after administration of DSB (1,000 mg twice a day [BID]) for 10 days (26), which is slightly less than the EC_50_ values (7.55 to 12.41 μM) determined in HIEs. Further *in vivo* studies are needed to determine the optimal treatment regimen of DSB that elicits anti-HuNoV activity with no toxicity in a clinical setting.

In summary, through the screening of an antiviral compound library, we identified DSB as a novel HuNoV inhibitor that warrants further clinical investigation. To our knowledge, this was the first time *bona fide* anti-HuNoV agents have been identified using the HIE culture system. Our study also sheds light on the usefulness of the HIE platform for investigation of anti-HuNoV agents and/or host factors regulating HuNoV infection, which will contribute to better understanding of the HuNoV life cycle and to the development of vaccine and antiviral regimens.

## MATERIALS AND METHODS

### Cells.

Human intestinal enteroid (HIE) J2 and J3 lines, established from jejunal biopsy specimens of secretor-positive adults (5), were provided from Baylor College of Medicine under a material transfer agreement. The study protocol was approved by the Review Board of the National Institute of Infectious Diseases in Japan. Wnt3a-producing cells were kindly provided by the Baylor College of Medicine. R-spondin- and Noggin-producing cells were kindly provided by Calvin Kuo (Palo Alto, CA) and Gijs van den Brink (University of Amsterdam, Netherlands), respectively. HIEs were grown as multilobular, 3-dimensional (3D) cultures in Matrigel and were maintained in complete medium with growth factors [CMGF(+)] or IntestiCult organoid growth medium (human) (Veritas) as previously described (5, 10, 19). Monkey kidney cell line MA104 was maintained in Dulbecco’s modified Eagle’s medium (DMEM) supplemented with 10% fetal bovine serum (FBS), 100 U/ml penicillin, and 100 μg/ml streptomycin. VeroE6/TMPRSS2 (JCRB1819, VeroE6 cell overexpressing the transmembrane protease, serine 2 [TMPRSS2]) (21) was purchased from JCRB Cell Bank (Osaka, Japan) and was maintained in DMEM supplemented with 10% FBS, 100 U/ml penicillin, 100 μg/ml streptomycin, and 1 mg/ml G418 (Nacalai).

### Viruses.

Ten percent stool suspensions containing human norovirus (HuNoV) were prepared as described previously (5) and kept at −80°C before use. Human rotavirus A (RVA) Wa strain was propagated in MA104 cells in the presence of trypsin. Severe acute respiratory syndrome coronavirus 2 (SARS-CoV-2), 2019-nCoV/Japan/TY/WK-521/2020 strain (WK-521), was isolated previously (21) and propagated in VeroE6/TMPRSS2 cells. Virus titer of the SARS-CoV-2 was determined by 50% tissue culture infectious dose (TCID_50_) assay in VeroE6/TMPRSS2 cells (21).

### Compounds.

An antivirus compound library (326 compounds, 10 mM solution in DMSO, L1700) was purchased from TargetMol. To validate antiviral effect, 3 individual dasabuvir (DSB) stocks were purchased from different companies (catalog no. T3489, TargetMol; catalog no. S5402, Selleck; and catalog no. 18482, Cayman Chemical). 2′-C-methylcytidine (2-CMC; catalog no. 22887) was purchased from Cayman Chemical. Remdesivir (RDV; catalog no. S8932) was purchased from Selleck.

### Infection of HIEs with viruses.

Virus infection in differentiated two-dimensional (2D) HIE monolayers was performed following a previously described protocol with minor modification (5, 10, 19). Briefly, 3D J2 (passages 19 to 35) or J3 (passages 25 to 27) HIE lines were dissociated with TrypLE Express (Thermo Fisher) into single cells, after which they were seeded onto collagen IV-coated 96-well plates at the number of approximately ∼10^5^ cells/well in CMGF(+) or IntestiCult medium supplemented with ROCK inhibitor Y-27632 (10 μM; Sigma) for 2 days. After 2 days, the cells typically reach ∼100% confluence. The medium was then removed, and the cells were maintained in the differentiation medium for another 2 days.

For HuNoV infection, the J2 and J3 HIE monolayers were inoculated with 10% stool filtrate containing 4.3 × 10^5^ genome equivalents (GEs) of GII.3 [GII.P21] (TCH04-577) (5, 12) or GII.4 [GII.P16] HuNoV in the presence of 500 μM glycochenodeoxycholic acid (GCDCA), which is required for GII.3 HuNoV infection and promotes GII.4 infection (5, 12). For RVA infection, the J2 HIE monolayers were inoculated with the Wa strain at 2.48 × 10^8^ GEs/well that were pretreated with 10 μg/ml trypsin (Sigma) for 1 h at 37°C to activate the VP4 spike protein (27). For SARS-CoV-2 infection, the J2 monolayers were incubated with WK-521 at 8.1 × 10^8^ GEs/well. After 1 h of incubation at 37°C, the cells were washed twice with complete medium without growth factors [CMGF(−)] to remove unbound viruses. The cells were then incubated with differentiation medium in the presence (HuNoV) or absence (RVA and SARS-CoV-2) of GCDCA until 20 to 24 h postinfection (hpi). The indicated compounds or DMSO were added to the medium throughout the infection process. The final concentration of DMSO was 0.5%. The cells and medium were then harvested and subjected to RNA extraction.

### RNA extraction and RT-qPCR.

Total RNA was extracted from the infected cells and/or media using the Direct-zol RNA MiniPrep kit (Zymo Research) according to the manufacturer’s instruction. RT-qPCR analysis to determine viral RNA GEs of HuNoV, RVA, and SARS-CoV-2 was performed using TaqMan Fast virus one-step master mix (Thermo Fisher) and specific primer/probe sets as described previously (5, 28, 29).

### Cell viability assay.

Cytotoxicity or cell viability upon compound treatment was determined using the Cytotoxicity LDH assay kit-WST (Dojindo) or the CellTiter-Glo luminescent cell viability assay (Promega), respectively, according to the respective manufacturer’s instructions.

### Immunofluorescence assay.

Confluent VeroE6/TMPRSS2 cells cultured in 96-well plate (CellCarrier-96 Ultra; Perkin Elmer) were infected with SARS-CoV-2 at a multiplicity of infection (MOI) of 0.003 for 20 h at 37°C. The infected cells were then fixed with 4% paraformaldehyde in Dulbecco’s phosphate-buffered saline (D-PBS) for 30 min and permeabilized with 0.2% Triton X-100 in D-PBS for 15 min. The cells were stained for SARS-CoV-2 spike (S) protein using rabbit anti-SARS-CoV-2 spike receptor-binding domain monoclonal antibody (1:3,000, clone HL1003, catalog no. GTX635792; GeneTex) followed by goat anti-rabbit IgG Alexa Fluor 488 (1:1,000; Life Technologies). Cell nuclei were stained with 1 μg/ml 4′,6-diamidino-2-phenylindole (DAPI) solution (Dojindo). The cells were then imaged using the Operetta CLS High-Content Analysis System (Perkin Elmer) and the percentage of SARS-CoV-2-positive cells (infectivity) in each well were calculated by counting SARS-CoV-2 S- and DAPI-positive cells using Harmony software (Perkin Elmer).

### Data analysis and statistics.

All experiments except for compound screening (Fig. 1 and Fig. S1 in the supplemental material), were performed at least two times with more than two technical replicates, and results are shown as mean ± SD (*n* ≥ 4). Statistical analysis was performed with analysis of variance (ANOVA) followed by Dunnett’s multiple-comparison test or two-tailed Student’s *t* test using GraphPad Prism 9 software. *P* values of <0.05 was considered statistically significant. A dose response curve was created by a nonlinear regression model, and the 50% effective concentration (EC_50_) and the cytotoxic concentration (CC_50_) were calculated using GraphPad Prism 9 software.
