# Supplementary figures and images for: Dasabuvir Inhibits Human Norovirus Infection in Human Intestinal Enteroids
Source: mSphere. 2021 Nov 3;6(6):e00623-21. doi: 10.1128/mSphere.00623-21 (PMC8565515; doi:10.1128/mSphere.00623-21)

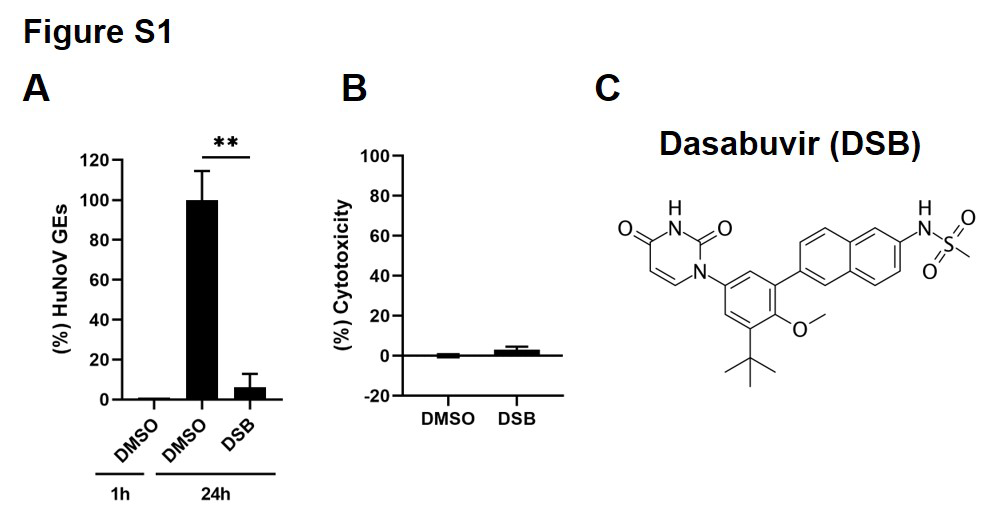

Supplement: FIG S1 [file msphere.00623-21-sf001.tif]

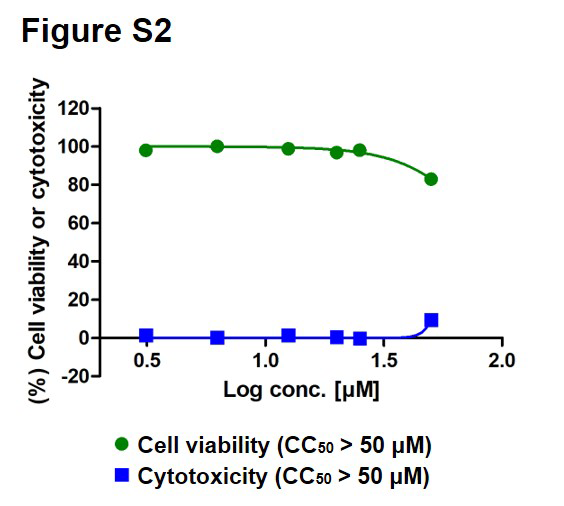

Supplement: FIG S2 [file msphere.00623-21-sf002.tif]

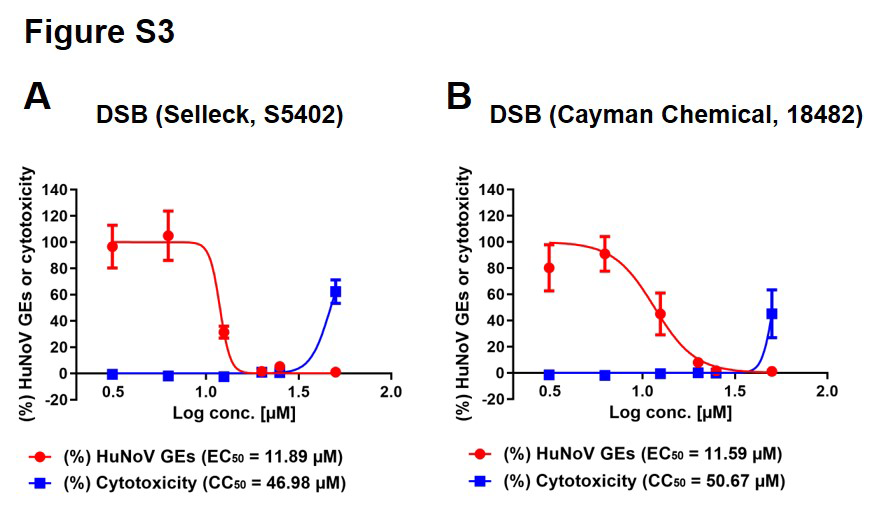

Supplement: FIG S3 [file msphere.00623-21-sf003.tif]

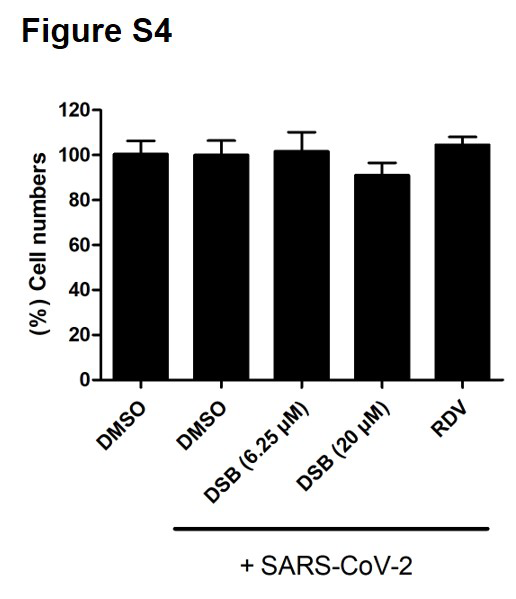

Supplement: FIG S4 [file msphere.00623-21-sf004.tif]
